# Maternal and perinatal outcomes of asylum seekers and undocumented migrants in Europe: a systematic review

**DOI:** 10.1093/eurpub/ckz042

**Published:** 2019-05-16

**Authors:** Noor C Gieles, Julia B Tankink, Myrthe van Midde, Johannes Düker, Peggy van der Lans, Catherina M Wessels, Kitty W M Bloemenkamp, Gouke Bonsel, Thomas van den Akker, Simone Goosen, Marcus J Rijken, Joyce L Browne

**Affiliations:** 1 Julius Global Health, Julius Center for Health Sciences and Primary Care, University Medical Center Utrecht, Utrecht, The Netherlands; 2 Research Department, Médecins du Monde/Dokters van de Wereld, Amsterdam, The Netherlands; 3 Department of Gynaecology and Obstetrics, Hospital Twente ZGT/MST, Enschede, The Netherlands; 4 Dutch Working Party on International Safe Motherhood and Reproductive Health, Amsterdam, The Netherlands; 5 Department of Obstetrics, Birth Centre Wilhelmina Children Hospital, Division Women and Baby, University Medical Center Utrecht, Utrecht, The Netherlands; 6 Department of Obstetrics, Leiden University Medical Center, Leiden, The Netherlands; 7 Netherlands Association of Community Health Services, Utrecht, The Netherlands

## Abstract

**Background:**

Asylum seekers (AS) and undocumented migrants (UM) are at risk of adverse pregnancy outcomes due to adverse health determinants and compromised maternal healthcare access and service quality. Considering recent migratory patterns and the absence of a robust overview, a systematic review was conducted on maternal and perinatal outcomes in AS and UM in Europe.

**Methods:**

Systematic literature searches were performed in MEDLINE and EMBASE (until 1 May 2017), complemented by a grey literature search (until 1 June 2017). Primary research articles reporting on any maternal or perinatal outcome, published between 2007 and 2017 in English/Dutch were eligible for inclusion. Review protocols were registered on Prospero: CRD42017062375 and CRD42017062477. Due to heterogeneity in study populations and outcomes, results were synthesized narratively.

**Results:**

Of 4652 peer-reviewed articles and 145 grey literature sources screened, 11 were included from 4 European countries. Several studies reported adverse outcomes including higher maternal mortality (AS), severe acute maternal morbidity (AS), preterm birth (UM) and low birthweight (UM). Risk of bias was generally acceptable, although the limited number and quality of some studies preclude definite conclusions.

**Conclusion:**

Limited evidence is available on pregnancy outcomes in AS and UM in Europe. The adverse outcomes reported imply that removing barriers to high-quality maternal care should be a priority. More research focussing on migrant subpopulations, considering potential risk factors such as ethnicity and legal status, is needed to guide policy and optimize care.

## Introduction

As migration into Europe has increased in the past decade, understanding the relation between migration and health outcomes can guide appropriate policy responses including improved healthcare provision.^1^^,^^2^ The high number of women and children among migrant populations requires a specific evaluation of maternal and perinatal health outcomes.^3^

Compared with host-country populations (HP), poorer as well as better pregnancy outcomes have been reported in migrants, the latter often being referred to as the ‘healthy migrant effect’.^4–10^ These divergent outcomes are a result of an interplay of determinants before migration, *en route* and after reaching the resettlement country.^11–13^ Disaggregating data on subpopulations of migrants may reveal why some migrant women have worse pregnancy outcomes than others.^14–16^ One way to differentiate migrants is by legal status, which determines many of their rights, social opportunities and access to healthcare.^17^ Through these factors, asylum seekers (AS) and undocumented migrants (UM) may face particular risks during pregnancy.^16^^,^^18–20^ Reviews on pregnancy outcomes in these groups are scarce and inconclusive, possibly as a result of heterogeneous study populations.^21–23^ This systematic review aims to provide an overview of maternal and perinatal outcomes in AS and UM in the past decade in Europe.

## Methods

### Protocol and registration

A protocol for each study population was initially registered with the international prospective register of systematic reviews: PROSPERO (AS: CRD42017062477 and UM: CRD42017062375). These protocols were based on PRISMA guidelines and merged after registration. To allow for a concise review focussed on health outcomes, we decided to deviate from the original registered protocols by only including quantitative studies into this review.

### Eligibility criteria

All studies reporting maternal, perinatal or neonatal outcomes (from 22 weeks of gestation up to 28 days postpartum) in AS or UM, available in English or Dutch, were eligible for inclusion. To study the effect of recent migration streams and policies in the European context, only studies from European countries published between 2007 and 2017 were included. As this review focussed on pregnancy outcomes, pregnancy-related findings such as induced abortion, unintended pregnancy and maternal infection during pregnancy were excluded.

Study populations were defined according to the International Organization of Migration (IOM, box 1):^24^

**Table:** Box 1 Definition of study populations

**Asylum seeker:** ‘A person who seeks safety from persecution or serious harm in a country other than his or her own and awaits a decision on the application for refugee status under relevant international and national instruments’.
**Undocumented migrant:** (…) ‘people whose entry, stay or work in a country is without the necessary authorization or documentation under immigration regulations’.

### Information sources and search

The systematic literature search was conducted in two electronic databases: Pubmed/MEDLINE and EMBASE (date last search: 1 May 2017). Peer-reviewed articles were identified using pre-defined search (Title/Abstract) and indexing terms (MeSH/Emtree) (search syntax: Supplementary file S1). Bibliographies of relevant studies were screened for additional references that fitted eligibility criteria. As (non-)governmental organizations may publish reports on the study populations of interest, an extensive grey literature search was conducted (date last search: 1 June 2017; for search syntax: Supplementary file S2). Moreover, (non-)governmental organizations, research and policy experts in the field of AS and UM were approached with a request for grey literature (organizations contacted: Supplementary file S3; request for grey literature: Supplementary file S4).

### Study selection

Titles and abstracts of the two searches were screened by two researchers independently (AS: J.B.T. & J.L.B., UM: N.C.G. & C.M.W.) using the online screening programme for systematic reviews Rayyan.^25^ Potentially eligible studies were assessed in full-text. If full-text was not available, the corresponding author of the paper was contacted once by e-mail to request the full-text version or translation. Authors were also contacted in cases where the legal status of the study population was uncertain. Any disagreement over eligibility of studies was resolved through discussion among the researchers until consensus was reached.

### Data collection process and data items

Data were extracted using a piloted extraction form. Data extraction was performed by one review author (AS: J.B.T. and UM: N.C.G.). None of the review authors was blinded for journal or author details. In case of missing data, the corresponding authors were approached once by e-mail. Extracted data included setting, design, data source, study period, study population (including sample size and nationalities), reference population and maternal/perinatal outcomes.

### Risk of bias assessment

Studies were assessed for risk of bias using the Newcastle-Ottawa Scale (NOS) for risk of bias in cohort studies, based on three criteria (selection, comparability and outcome).^26^ The NOS was adapted to color-coding where orange and red refer to one and multiple negative scores on a sub-question, respectively.

### Summary measures

Where available, the odds ratio (OR) or risk ratio (RR) including a 95% confidence interval (95% CI) of an outcome was reported. Alternatively, when these outcome measures were not provided, the sample size, percentages and corresponding *P*-value were reported. If no statistical analysis had been conducted over results, only sample size and percentages were reported.

### Synthesis of results

Extracted data were evaluated to determine whether meta-analysis with pooled data was possible, as originally intended. However, given the heterogeneity of outcomes reported and the differences in study populations regarding nationality, sample size and setting, results could not be pooled into a meta-analysis. Data synthesis therefore provides a narrative review of maternal and perinatal outcomes.

## Results

### Study selection

**Figure 1:**
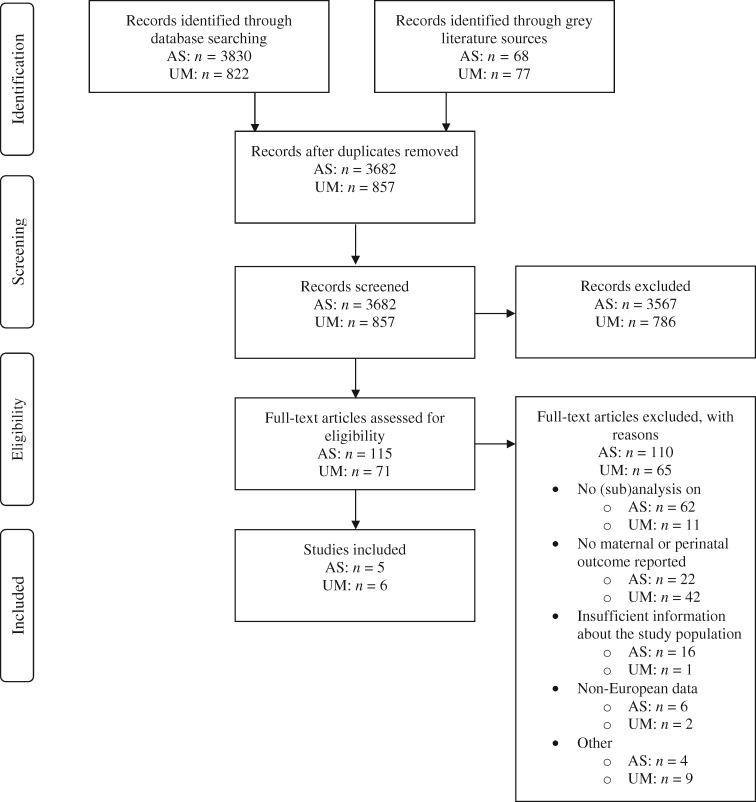
Flow diagram of study selection process in asylum seekers (AS) and undocumented migrants (UM)

The literature search generated 3682 unique results for AS and 857 for UM, of which 68 (AS) and 77 (UM) were grey literature sources. After screening on title/abstract, 115 (AS) and 71 (UM) manuscripts were screened in full-text. Full-text screening resulted in inclusion of 11 articles (AS: 5, all peer-reviewed, UM: 6, of which one grey literature source) (figure 1).

### Study characteristics

An overview of study characteristics is presented in table 1. Two included studies had prospective cohort designs, six were retrospective cohorts and three were cross-sectional studies. Studies were conducted in The Netherlands (AS: three, UM: two), Switzerland (AS: two, UM: one), Italy (UM: two) and the UK (UM: one).

**Table 1:** Study characteristics of studies reporting maternal or perinatal outcomes in asylum seekers and undocumented migrants (*n* = 11)

Author(s) (year)	Region/country	Setting	Research methods	Period	Study population (size)	Nationalities of study population (%)	Reference population (size)
**Asylum seekers**							
Kurth et al. (2010)^29^	Basel, Switzerland	University Hospital of Basel	Design: retrospective cohort, mixed-methods (qualitative and quantitative)	2000–03	AS attending women’s clinic (80; deliveries among them: 48)	Former Yugoslavia (50%), Africa (19%), Asia (16%), E-Europe (9%), other countries (6%)	Total patient population (6396)
			Data source: hospital demographic database semi-structured interviews medical records				
Van Oostrum et al. (2011)^28^	The Netherlands	Community health services for AS	Design: retrospective cohort	2002–05	AS (4327 deliveries)	W-, C- and S-Africa (22.5%), N-, E- and Horn of Africa (11.1%), C-, E- and S-Europe (26.8%), Middle East and S-W-Asia (31.1%), C-, E- and S-Asia (5.4%), other (3.2%)	Dutch population in 2002–05
			Data source: national mortality and birth registers				
Van Hanegem et al. (2011)^27^	The Netherlands	Maternity wards of all public hospitals	Design: retrospective cohort	2004–06	Pregnant AS with SAMM (40)	Africa (47.5%), Middle East (15.0%), Turkey (10.0%), Asia (12.5%), S-America (5.0%), Former USSR (5.0%), other non-Western (5.0%)	Non-Western migrants with SAMM (517) General Dutch population with SAMM (2512)
			Data source: national registry data on maternal morbidity birth registration by the Central Agency for the Reception of Asylum Seekers				
Goosen et al. (2015)^31^	The Netherlands	Community health services for AS	Design: cross-sectional	2000–08	HIV-positive AS who delivered (80)	Sub-Saharan Africa (98.8%)	HIV-negative AS who delivered (4774)
			Data source: electronic medical records from the community health services for AS				
Ratcliff et al. (2015)^30^	Geneva, Switzerland	Midwifery practice	Design: cross-sectional	2006–14	Migrant women with precarious legal status attending antenatal education programme (118)	AS and refugees: Africa (37%), Asia (13%), the Middle East (17%), C- or E-Europe (7%), N-Africa (3%), other (14%)	Migrant women with non-precarious legal status attending antenatal education programme (110)
			Data source: questionnaires Edinburgh Postnatal Depression Scale midwives' observations and participants’ self-report				
						Undocumented: Latin America (19%)	
**Undocumented migrants**							
Wolff et al. (2008)^34^	Geneva, Switzerland	Midwifery healthcare unit in collaboration with the Geneva University Hospital	Design: prospective cohort	2005–06	Pregnant UM (161)	Bolivia (34.8%), Brazil (23%), Columbia (8.7%), Ecuador (6.2%), Peru (5.6%), Philippines (3.7%)	Pregnant women with legal residence permit (both migrants and locals) (233)
			Data source: questionnaires blood tests medical records				
Schoevers et al. (2009)^32^	The Netherlands	Support organizations, community networks and healthcare providers	Design: cross-sectional Data source: interviews	Unknown	Pregnant UM and undocumented migrant mothers (31 pregnancies, 22 deliveries in 27 women)	E-Europe (30%), Sub-Saharan Africa (21%), Turkey/Middle E-/N-Africa (12%), China/Mongolia (12%), Afghanistan/Iran (11%), Middle and S-America/Philippines (8%), Surinam (6%)	None
Fedeli et al. (2010)^36^	Veneto, Italy	Obstetric wards of acute care hospitals	Design: retrospective cohort Data source: hospital discharge records	2006–07	UM and a minority of migrants with a short-term residence (1870)	Unknown	Veneto residents with Italian citizenship (73 098) foreign citizenship (18 462)
de Jonge et al. (2011)^33^	Amsterdam and Rotterdam, The Netherlands	Primary care midwifery practices	Design: retrospective cohort Data source: medical records	2005–06	Pregnant UM (141)	All from non-Western European countries	Pregnant DM (141)
Shortall et al. (2015)^35^	London, UK	Drop-in healthcare unit, Doctors of the World	Design: prospective cohort study Data source: medical records semi-structured (telephone) questionnaires	2013–14	Pregnant migrants visiting the Doctors of the World drop-in clinic, the majority being UM (35)	Unknown	NA

Salmasi et al. (2015)^37^	Italy	All deliveries nation-wide	Design: retrospective cohort Data source: birth sample surveys from 2001 and 2003	2002–05	Newborns from UM (2344)	Worldwide, please refer to the appendix of Salmasi et al. (2015) for a full overview	DM with Italian nationality (4189) Women born in Italy (90 578)

Notes: Under ‘Nationalities of study population’: N, north(ern); E, east(ern); S, south(ern); W, west(ern); C, central; AS, asylum seekers; DM, documented migrants; UM, undocumented migrants.

Studies on AS mostly (*n* = 3) compared AS to HP.^27–29^ One study compared the HIV mother-to-child transmission rate in AS over time. UM and AS were compared with a group of migrants with long-term or permanent residence permits in another study.^30^ In studies on UM, two studies included the HP as a comparison group. In two other studies, UM were compared with documented migrants (DM; i.e. migrants with legal residence permit) alone. Two studies did not have a control group. One study with a control group did not apply statistical analysis to their data.

An overview of all maternal and perinatal outcomes in AS and UM is presented in table 2. In Supplementary file S5, the relative (dis)advantage of AS and UM per outcome is shown. Other than perinatal outcomes (up to 7 days postpartum), no neonatal outcomes were reported in included studies.

**Table 2:** Overview of maternal and perinatal outcomes in asylum seekers and undocumented migrants for each of the included studies

Author(s) (year)	Maternal outcomes	Perinatal outcomes
**Asylum seekers**		
Kurth et al. (2010)^29^	Mode of birth: no difference Spontaneous abortions: 8%^⋄^ Premature labour: 15%^⋄^ Prenatal bleeding: 11%^⋄^ Gestational diabetes: 9%^⋄^ Anaemia: 7%^⋄^	Preterm birth (35–37 weeks): 6% (*n* = 3/48)^⋄^ Mean birthweight (SD): 3470 g (556)^⋄^ Low birthweight (<2500 g): 2% (*n* = 1/48)^⋄^ Congenital malformations: 2% (*n* = 1/48)^⋄^ Intrauterine growth restriction: 7%^⋄^
Van Oostrum et al. (2011)^28^	Maternal mortality ratio (deaths per 100 000 births): higher [AS vs. HP: rate ratio (95% CI) = 10.08 (8.02, 12.83)] Maternal mortality ratio (deaths per 100 000 births): higher [AS vs. Surinams/Antillians (ethnic group with highest maternal mortality ratio in The Netherlands): 69.33 vs. 35]	Perinatal mortality: no difference
Van Hanegem et al. (2011)^27^	Maternal deaths: none Incidence of SAMM (per 1000 births): higher [*n* = 369 711, AS vs. HP: 31.0 vs 6.8, RR (95% CI) = 4.5 (3.3, 6.1); AS vs. non-western DM: 31.0 vs 8.5, RR (95% CI) = 3.6 (2.6, 5.0)] SAMM (per 1000 births): higher [AS vs. non-Western migrants: 31.0 vs. 8.5, RR (95% CI) = 3.6 (2.6, 5.0)] –Inclusion categories for SAMM: ICU admission, uterine rupture, eclampsia/HELLP, major obstetric haemorrhage, miscellaneous (other types of SAMM)	NA
Goosen et al. (2015)^31^	NA	Mother-to-child transmission of HIV: 9.8% (*n* = 6/62)
Ratcliff et al. (2015)^30^	Antenatal depression (EPDS score): no difference (AS & UM vs. DM)	Obstetric complications: no difference –Complications considered: e.g. premature birth, infection, gestational diabetes, hypertension or pre-eclampsia, haemorrhage
**Undocumented migrants**		
Wolff et al. (2008)^34^	Mode of birth: no difference Complications during pregnancy, vaginal birth or postpartum: no difference –Pregnancy and vaginal birth complications considered: urinary infection, anaemia, risk of preterm birth, vaginal tear, retention of the placenta, pre-eclampsia, fever	Mean gestational age in weeks (SD): lower (*n* = 335, UM vs. DM: 38.9 (1.9) vs. 39.4 (1.4), *P* = 0.02) Preterm births (<37 weeks): no difference (*n* = 335, UM vs. DM: 9% vs. 4%, *P* = 0.09) Born in good health, born dead, transfer to the neonatology for serious health hazard, birth weight, low birth weight, APGAR scores and neonatal complications: no difference
Schoevers et al. (2009)^32^	Hypertension/toxemia during pregnancy: 6% (*n* = 2/31)^⋄^ Kidney problems during pregnancy: 3% (*n* = 2/31)^⋄^ Cervix insufficiency: 3% (*n* = 1/31)^⋄^	Preterm birth: 9% (*n* = 2/22)^⋄^ Caesarean section: 9% (*n* = 2/22)^⋄^ Prolonged labour: 5% (*n* = 1/22)^⋄^ Multiple handicapped foetus: 5% (*n* = 1/22)^⋄^ Embryoma spine neonate: 5% (*n* = 1/22)^⋄^ Low birth weight (<2500 g): 14% (*n* = 3/22)^⋄^ Foetal distress 5% (*n* = 1/22)^⋄^ Birth trauma: 5% (*n* = 1/22)^⋄^
Fedeli et al. (2010)^36^	Birth via caesarean section: (*n* = 93 430, UM vs. DM vs. HP: 19.5% vs. 26.9% vs. 29.5%)^⋄^ Antepartum hospitalizations per birth: (*n* = 93 430, UM vs. DM vs. HP: 0.24 vs. 0.21 vs. 0.1)^⋄^ Miscarriages per birth: (*n* = 93 430, UM vs. DM vs. HP: 0.35 vs. 0.15 vs. 0.1)^⋄^	NA
de Jonge et al. (2011)^33^	Intervention during labour: no difference. Intervention during labour: no difference. –Interventions considered: induction, augmentation, vacuum, forceps, caesarean section Referral for failure to progress in labour: lower [*n* = 226, UM vs. DM: 7% vs. 24%, OR (95% CI) = 0.40 (0.16, 0.98)] Anaemia: no difference Neonatal admission at maternal indication: no difference	Perinatal mortality (>22 weeks): no difference Gestational age at birth in weeks: no difference Preterm birth (<37 weeks): higher [*n* = 226, UM vs. DM: 12.6% vs. 3.1%, OR (95% CI) = 4.59 (1.43, 14.72)] Foetal distress: no difference Weight of babies born at term: no difference Low birth weight (<2500 g): higher [*n* = 226, UM vs. DM: 14% vs. 6%, OR (CI 95%) = 3.51 (1.30, 9.52)] Neonatal admissions for prematurity/SGA: higher [*n* = 226, UM vs. DM: 12% vs. 3%, OR (95% CI) = 6.17 (1.69, 22.52)] Neonatal admissions for poor neonatal condition: no difference
Shortall et al. (2015)^35^	Mode of birth: elective caesarean section: 2.9% (*n* = 1/35) emergency caesarean section: 29.4% (*n* = 10/35) instrumental birth: 5.9% (*n* = 2/35) spontaneous vaginal birth: 58.8% (*n* = 20/35)^⋄^	Birth at term: 43%^⋄^ Postterm birth: 37%^⋄^ Preterm birth: 14%^⋄^ Perinatal mortality: 5.5%; (*n* = 2/35)^⋄^
Salmasi et al. (2015)^37^	NA	Low birth weight decreased with 1.2–2.7% (*P* < 0.05) when UM had become DM

No difference: AS/UM have been compared with HP/DM, no statistical differences found. Higher/lower: significantly higher/lower results reported for study population (AS/UM) as compared with control population (HP/DM) on this outcome. ^⋄^No statistical analysis available on this outcome. Notes: APGAR, Appearance, Pulse, Grimace, Activity, Respiration; AS, asylum seekers; DM, documented migrants; EPDS, Edinburgh Postnatal Depression Scale; HELLP, haemolysis, elevated liver enzymes, low platelet count; HP, host-country population(s); ICU, intensive care unit; SAMM, Severe Acute Maternal Morbidity; SGA, Small for Gestational Age; UM, undocumented migrants.

### Narrative summary of results

#### Asylum seekers

##### Maternal outcomes

Maternal mortality ratio was reported 10-fold higher for AS compared with the Dutch population [RR (95% CI) = 10.08 (8.02–12.83)].^28^ In another Dutch study, severe acute maternal morbidity (SAMM; defined as ICU admission, uterine rupture, eclampsia/HELLP syndrome, major obstetric haemorrhage or miscellaneous) occurred at a rate 4.5 times higher in AS compared with the HP [RR (95%CI) = 4.5(3.3–6.1)], and 3.6 times higher compared with other non-Western DM [RR (95%CI) = 3.6 (2.6–5.0)].^27^ In Switzerland, no differences were found for a composite of obstetric complications—including pre-eclampsia and postpartum haemorrhage—between undocumented or refugee women with a temporary residence permit and women with long-term or permanent residence permits. The same study found high overall antenatal depression scores but found no significant difference between the study groups.^30^

With regards to mode of birth, no differences were found for vaginal and instrumental birth and caesarean section rates in a small Swiss study (*n* = 48 deliveries).^29^ In The Netherlands, no differences were reported in induction of labour or caesarean section rates among AS, non-Western DM and native Dutch women who experienced SAMM (*n* = 3087 cases).

##### Perinatal outcomes

Perinatal outcomes were reported in some studies, but these did not compare AS to a reference group. In one of these studies, mother-to-child transmission rate of HIV was 9.8% among AS in The Netherlands (*n* = 80 births to HIV-positive asylum seeking women). All children who were HIV-positive at birth were born before the implementation of universal HIV screening in 2004.^31^

#### Undocumented migrants

##### Maternal outcomes

Several studies reported maternal outcomes in UM.^32–36^ A study from Italy found a higher ratio of spontaneous abortions/miscarriages to births compared with DM and to Italian residents in a large study from Italy, although no statistical analysis was performed in this study (*n* = 93 430, UM vs. DM vs. HP: 0.35 vs. 0.15 vs. 0.16).^36^ The same study reported higher ratios of antepartum hospitalizations per birth for UM (*n* = 93 430, UM vs. DM vs. HP: 0.24 vs. 0.21 vs. 0.18). A study from Switzerland showed no significant differences compared with DM in pre-eclampsia, gestational diabetes, anaemia, prenatal bleeding, urinary tract infection and risk of preterm birth.^34^ A study from The Netherlands reported rates of pre-eclampsia, cervix insufficiency and kidney problems during pregnancy but did not include a control group.^32^

Five studies reported findings regarding mode of birth and had conflicting results.^32–36^ Rates of vaginal birth and interventions during labour (including induction, vacuum/forceps and caesarean section) were similar for UM and DM in The Netherlands.^33^ This was confirmed in the Swiss study, where spontaneous birth, forceps, vacuum and caesarean sections were similar among UM and DM.^34^ A lower caesarean section rate was reported in Italy [UM: 19.5% (*n* = 365/1870) vs. DM: 26.9% (*n* = 4966/18 462) vs. HP: 29.5% (*n* = 21 564/73 098)], whereas a high rate of emergency caesarean sections was reported by a small study published by Doctors of the World in the UK [29% (*n* = 10/35)].^35^^,^^36^

##### Perinatal outcomes

Regarding perinatal outcomes, UM were more likely to give birth prematurely compared with DM in The Netherlands [*n* = 226, UM vs. DM: 12.6% vs. 3.1%, OR (95% CI) = 4.59 (1.43, 14.72)].^33^ The Swiss study showed no significant differences but reported a trend of higher preterm birth rate as well (*n* = 335, UM vs. DM: 9% vs. 4%, *P* = 0.09).^34^ Regarding birthweight, a higher rate of low birthweight (<2500 g) was found in The Netherlands [*n* = 226, UM vs. DM: 15% vs. 5%, OR (CI 95%) = 3.51 (1.30, 9.52)], although birthweight for babies born at term was not significantly different.^33^ The Swiss study reported a higher rate of low birthweight in UM, but this difference was not significant (*n* = 335, UM vs. DM: 4.7% vs. 2.6%, *P* = 0.24).^34^ Finally, a large-scale study (*n* = 2344 newborns) in Italy showed that the number of babies born with low birthweight decreased when UM became documented as a result of a change in law (1.2% vs. 2.7%, *P* < 0.05).^37^

The overall health of the neonate, including being born in good health, stillbirth, APGAR scores and transfer to the neonatology ward for serious health hazard was not different between UM and DM in the Swiss study.^34^ In the same study, neonatal complications (not further defined) showed a trend towards worse outcomes in UM vs DM: (*n* = 335, UM vs. DM: 2.8% vs. 6.6%, *P* = 0.07).^34^ The number of newborns admitted to the hospital for poor neonatal condition was similar for UM and DM in The Netherlands.^33^

### Risk of bias of individual studies

The risk of bias assessment for individual studies was conducted using the NOS for cohort and cross-sectional studies (risk of bias: Supplementary file S6). Risk of bias was low in the majority of studies (*n* = 6/11) included. Four studies had moderate, and one had high risk of bias. Risk of bias emerged from diverse sources, such as selective facility-based sampling methods, small sample sizes and the lack of a control group in two studies on UM. A number of other studies had control groups that were not adequately described or that differed from the study population in important population characteristics such as age, ethnicity or time spent in the country (*n* = 4/11). One study reported data on mother-to-child transmission rate of HIV in AS but did not include a reference group for this outcome.^31^ Most studies used hospital or registry data (*n* = 9/11), while some depended (partially) on self-reported outcomes (*n* = 4/11).

## Discussion

This review systematically assessed the literature on maternal and perinatal outcomes in AS and UM in Europe published in the past decade. An overall lack of high-quality quantitative research was identified, and available studies only covered four European countries (Italy, The Netherlands, Switzerland and the UK). The studies showed no consistent pattern of adverse outcomes, although none of the studies reported a healthy migrant effect (i.e. more favourable outcomes) in AS or UM compared with host-country populations. Some well-designed studies reported adverse findings, including a higher maternal mortality and severe maternal morbidity rates among AS in The Netherlands compared with host-country populations.^27^^,^^28^ In UM, higher preterm birth and low birth weight rates were reported compared with documented migrants.^33^^,^^37^

The heterogeneity of maternal and perinatal health findings of this review suggests that legal status (asylum seeking or undocumented) is part of a complex interplay of potential risk and resilience factors.^12^^,^^38^ Among AS and UM, women with a refugee background may be particularly at risk of adverse outcomes.^6^^,^^16^^,^^20^^,^^39^^,^^40^ Additional risk factors for AS include short length of residence, low socio-economic status and language barriers.^27^ UM face increased risk as a result of precarious material and social conditions, such as poor housing and fear of deportation as well as stringent regulations limiting employment opportunities and healthcare access.^17^^,^^18^ Maternal country of origin is another key characteristic considered a potential risk factor for adverse maternal and perinatal health outcomes.^16^^,^^30^^,^^41^ Conversely, positive integration policies, becoming documented and adopting host-country nationality can improve maternal and perinatal health outcomes.^4^^,^^37^^,42,^^43^

The rather low number of studies included in this review illustrates general issues in research on ‘migrants’—a poorly demarcated group of which variables such as ethnicity, length of residence and legal status are not standardly registered or reported.^6^^,^^16^ The aim of this review was to strictly consider migrants with temporary or uncertain residence permits (AS) or no residence permits at all (UM). Although these stringent inclusion criteria allowed us to specifically explore the role of legal status as a determinant in maternal/perinatal health care, it also resulted in the exclusion of high-quality studies if insufficient information was available about the legal status of the study populations. Some of these studies included women from war-affected countries or with short length of residence in the destination country, who could in fact be AS or UM. Among these, Bakken et al. (2015) reported increased risk of adverse obstetric outcomes in Somalians but not in women from other conflict-zone countries.^44^ Liu et al. (2014) showed that war refugees (with unknown legal status) had increased risk of preterm birth in their first year in Sweden, compared with the following year.^45^ Other studies included AS or UM as part of the study population, but did not present a sub-analysis on these groups, hence did not meet inclusion criteria.^45^ The lack of such analyses reflects legal, social and administrative barriers to clinical registration of migration-related determinants in national databases.

Several limitations should be considered in interpreting the findings of this review. Meta-analysis could not be performed due to heterogeneity across studies in design and methods. Narrative synthesis of results relied on studies with several methodological limitations, such as the lack of a control group in some studies.^29^^,^^32^^,^^35^ Caution is warranted when interpreting non-significant results from studies whose sample size was not powered to detect differences for specific outcomes. Particularly in studies on UM, study and control populations often differed in important characteristics such as ethnicity, nationality and length of residence. Findings were not always controlled for confounding factors, such as low birthweight for prematurity. Moreover, as UM were mostly compared with documented migrants, differences to host-country population(s) may be underestimated. Bias may also arise from the comparison of migrant groups across different destination countries. Studies comparing AS to UM do, to the best of our knowledge, not exist. Finally, countries with a high recent influx of migrants (such as Greece and the Balkan countries) as well as ethnicities most prevalent in these migrant groups (such as Syrian, Afghan and Iraqi) were not covered in included studies.^2^

In parallel, this review has important strengths. The systematic search involved academic and grey literature sources, including a search through experts in the field. This strategy recognized the substantial expertise of non-governmental and other support organizations that may not publish their findings through academic channels. The extensive search guaranteed the inclusion of a wide range of pregnancy outcomes. The focus on European studies published between 2007 and 2017 allowed for the consideration of migratory movements in a specific geographical context and time-frame.

The findings and limitations of available studies translate into several recommendations for future research and policy. To address or compare findings in specific migrant subgroups, a list of indicators to be included in standardized perinatal data collection was developed in a Delphi study by the Reproductive Outcomes and Migration (ROAM) collaboration.^46^ ROAM recommended characteristics to be collected include country of origin and length of residence. Legal status was also considered to be important, yet less feasible for clinical registration.

Furthermore, several outcomes that were not considered in the current review deserve future research attention, such as the prevalence of maternal infections during pregnancy, unintended pregnancy and induced abortions.^47–49^ As higher adverse maternal mental health outcomes have been reported for AS outside of Europe, there is a need for such enquiries in Europe.^39^ Similarly, barriers in access to maternal and reproductive healthcare services, for contraceptives and antenatal care, exist and require further evaluation.^32,^^50^ The impact of European migration policies on maternal and perinatal outcomes could provide critical insights, e.g. by using the MIPEX-index, which assesses migrant integration policies across all EU Member States.^52^ Beyond quantitative assessments, qualitative studies and collaboration with support organizations can provide insight into the specific needs and experiences of AS and UM, and guide culturally sensitive care provision.^51,^^53^ Audit studies are an important tool in the prevention of maternal and perinatal mortality or severe morbidity.^54,^^55^ Finally, as emphasized by the recent report of the Guttmacher-Lancet Commission, policy should protect maternal and neonatal health by ensuring access to sexual and reproductive health and rights for all, including vulnerable groups such as AS and UM.^56^

## Conclusion

In conclusion, this systematic review highlights the need for standardized high-quality quantitative research on maternal and perinatal outcomes in AS and UM in Europe. Available studies are limited in number, heterogeneous in design and have several methodological limitations. Higher rates of maternal mortality and severe morbidity among AS have been reported, as well as higher rates of preterm birth and low birthweight among UM. These adverse findings necessitate further evaluation of the role of legal status and other factors affecting the health of migrant women and their newborns in European countries. In future clinical registration and research into maternal and perinatal health, disaggregating data for migrant subpopulations facing different health determinants would help to disentangle the mechanisms underlying adverse outcomes or a healthy migrant effect. To guarantee safe motherhood for coming generations of people entering or remaining in Europe, reducing the barriers to optimal maternity care should be a priority in migrant health research, policy and practice.

## 

*Conflicts of interest*: None declared.

**Key points**

- Asylum seekers and undocumented migrants are subpopulations of migrants who face specific negative health determinants that put them at risk of adverse maternal and perinatal outcomes.
- This review identified a lack of research on asylum seekers and undocumented migrants. The available studies often have methodological limitations.
- Adverse outcomes reported for asylum seekers include a higher maternal mortality and severe acute maternal morbidity compared with the host country’s population.
- Adverse outcomes reported for undocumented migrants include more preterm birth and low birth weight in babies from undocumented migrants compared with documented migrants.
- Clinical registration of migration-related health determinants and studies that disaggregate data for migrant subpopulations could provide insight into risk factors for adverse outcomes and how to address these in policy and practice.

## Supplementary Material

ckz042_Supplementary_Files
