# Supplementary material for: Maternal and perinatal outcomes of asylum seekers and undocumented migrants in Europe: a systematic review
Source: Eur J Public Health. 2019 May 16;29(4):714–23. doi: 10.1093/eurpub/ckz042 (PMC6734941; doi:10.1093/eurpub/ckz042)
Supplement: ckz042_Supplementary_Files [file ckz042_supplementary_files.docx]

**Supplementary files**

Table of contents

File 1. Search syntax asylum seekers and undocumented migrants

File 2. Grey literature search strategy

File 3. Organisations contacted with request for grey literature

File 4. Request for grey literature

File 5. Overview of reported outcomes and the direction of findings in each study

File 6. Risk of bias assessment according to t*he Newcastle-Ottawa Scale (NOS)*

File 7. Additional references (41-56)

**File 1. Search syntax asylum seekers and undocumented migrants**

**Asylum seekers:**

**Search Pubmed/MEDLINE (25^th^ of April 2017) (search syntax was adapted to Embase database)**

**#1 AND #2 AND (#3 OR #4)**

#1

Asylum[Title/Abstract] OR refugee*[Title/Abstract] OR migrant*[Title/Abstract] OR immigrant*[Title/Abstract] OR immigration[Title/Abstract] OR migration[Title/Abstract] OR transients and migrants[MeSH Terms] OR refugees[MeSH Terms]

#2

Europe[MeSH Terms] OR Albania*[Title/Abstract] OR Andorra*[Title/Abstract] OR Austria*[Title/Abstract] OR Belarus*[Title/Abstract] OR Belgium[Title/Abstract] OR Bosnia[Title/Abstract] OR Bulgaria*[Title/Abstract] OR Croatia*[Title/Abstract] OR Cyprus[Title/Abstract] OR Czech*[Title/Abstract] OR Denmark[Title/Abstract] OR England[Title/Abstract] OR Estonia*[Title/Abstract] OR Faroe*[Title/Abstract] OR Faeroe*[Title/Abstract] OR Finland[Title/Abstract] OR France[Title/Abstract] OR Germany[Title/Abstract] OR Gibraltar*[Title/Abstract] OR Greece[Title/Abstract] OR Great-Britain[Title/Abstract] OR Great Britain[Title/Abstract] OR Holland[Title/Abstract] OR Hungary[Title/Abstract] OR Iceland*[Title/Abstract] OR Ireland[Title/Abstract] OR Northern Ireland[Title/Abstract] OR Italy[Title/Abstract] OR Kosovo*[Title/Abstract] OR Latvia*[Title/Abstract] OR Liechtenstein*[Title/Abstract] OR Lithuania*[Title/Abstract] OR Luxembourg*[Title/Abstract] OR Luxemborg*[Title/Abstract] OR Macedonia*[Title/Abstract] OR Malta*[Title/Abstract] OR Moldavia*[Title/Abstract] OR Monaco[Title/Abstract] OR Montenegro[Title/Abstract] OR Netherlands[Title/Abstract] OR Norway[Title/Abstract] OR Poland[Title/Abstract] OR Portugal[Title/Abstract] OR Romania*[Title/Abstract] OR San Marino[Title/Abstract] OR Scotland[Title/Abstract] OR Serbia*[Title/Abstract] OR Slovenia*[Title/Abstract] OR Slovakia*[Title/Abstract] OR Spain[Title/Abstract] OR Sweden[Title/Abstract] OR Switzerland[Title/Abstract] OR United Kingdom[Title/Abstract] OR UK[Title/Abstract] OR Ukraine[Title/Abstract] OR Vatican*[Title/Abstract] OR Wales[Title/Abstract] OR Belgian[Title/Abstract] OR Bosnian[Title/Abstract] OR British[Title/Abstract] OR Cypriot[Title/Abstract] OR Danish[Title/Abstract] OR Dutch[Title/Abstract] OR English[Title/Abstract] OR Finnish[Title/Abstract] OR French[Title/Abstract] OR German[Title/Abstract] OR Greek[Title/Abstract] OR Hungarian[Title/Abstract] OR Irish[Title/Abstract] OR Italian[Title/Abstract] OR Kosovar*[Title/Abstract] OR Maltese[Title/Abstract] OR Monacan[Title/Abstract] OR Norwegian[Title/Abstract] OR Polish[Title/Abstract] OR Portuguese[Title/Abstract] OR Scottish[Title/Abstract] OR Spanish[Title/Abstract] OR Swedish[Title/Abstract] OR Swiss[Title/Abstract] OR Ukrainian[Title/Abstract] OR Welsh[Title/Abstract] OR Europe*[Title/Abstract] OR EU[Title/Abstract] OR Benelux[Title/Abstract] OR Western-Europe*[Title/Abstract] OR Western Europe*[Title/Abstract] OR Eastern-Europe*[Title/Abstract] OR Eastern Europe*[Title/Abstract] OR Scandinavia*[Title/Abstract] OR Balkan[Title/Abstract] OR Nordic[Title/Abstract] OR Baltic States[Title/Abstract] OR Mediterranean[Title/Abstract] OR Western countr*[Title/Abstract] OR Destination countr*[Title/Abstract] OR High-income countr*[Title/Abstract] OR High-immigration setting*[Title/Abstract] OR Host countr*[Title/Abstract] OR Immigrant-receiving countr*[Title/Abstract] OR migrant-receiving countr*[Title/Abstract]

#3

Pregnanc*[Title/Abstract] OR pregnant[Title/Abstract] OR mother*[Title/Abstract] OR matern*[Title/Abstract] OR conception[Title/Abstract] OR delivery[Title/Abstract] OR partus[Title/Abstract] OR labor[Title/Abstract] OR labour[Title/Abstract] OR childbirth[Title/Abstract] OR cesarean[Title/Abstract] OR caesarean[Title/Abstract] OR section[Title/Abstract] OR puerper*[Title/Abstract] OR breastfeeding[Title/Abstract] OR lactation[Title/Abstract] OR reproductive[Title/Abstract] OR sexual health[Title/Abstract] OR antenatal[Title/Abstract] OR prenatal[Title/Abstract] OR postnatal[Title/Abstract] OR perinatal[Title/Abstract] OR antepartum[Title/Abstract] OR peripartum[Title/Abstract] OR intrapartum[Title/Abstract] OR postpartum[Title/Abstract] OR post-partum[Title/Abstract] OR post birth[Title/Abstract] OR after birth[Title/Abstract] OR obstetric*[Title/Abstract] OR maternal health[MeSH Terms] OR maternal health services[MeSH Terms] OR obstetrics[MeSH Terms] OR maternal child nursing[MeSH Terms] OR pregnancy[MeSH Terms] OR postnatal care[MeSH Terms] OR prenatal care[MeSH Terms] OR perinatal care[MeSH Terms] OR Maternal-child nursing[MeSH Terms] OR reproductive health[MeSH Terms] OR Neonat*[Title/Abstract] OR newborn[Title/Abstract] OR infant*[Title/Abstract] OR baby[Title/Abstract] OR babies[Title/Abstract] OR congenital[Title/Abstract] OR fetus[Title/Abstract] OR fetal[Title/Abstract] OR Infant[MeSH Terms] OR Infant Health[MeSH Terms]

#4

SAMM[Title/Abstract] OR near miss[Title/Abstract] OR near-miss[Title/Abstract] OR abortion[Title/Abstract] OR miscarriage[Title/Abstract] OR ectopic OR preeclampsia[Title/Abstract] OR eclampsia[Title/Abstract] OR HELLP[Title/Abstract] OR placenta*[Title/Abstract] OR gestation*[Title/Abstract] OR hyperemesis gravidarum[Title/Abstract] OR PPH[Title/Abstract] OR haemorrhage[Title/Abstract] OR hemorrhage[Title/Abstract] OR hypovolem*[Title/Abstract] OR hysterectomy[Title/Abstract] OR sepsis[Title/Abstract] OR septic[Title/Abstract] OR malpresentation[Title/Abstract] OR breech[Title/Abstract] OR dystocia[Title/Abstract] OR chorioamnionitis[Title/Abstract] OR amniotic[Title/Abstract] OR endometritis[Title/Abstract] OR umbilical cord[Title/Abstract] OR birth weight[Title/Abstract] OR ICU admission[Title/Abstract] OR NICU admission[Title/Abstract] OR vaginal[Title/Abstract] OR genital[Title/Abstract] OR uterine[Title/Abstract] OR uterus[Title/Abstract] OR prematur*[Title/Abstract] OR preterm[Title/Abstract] OR pre-term[Title/Abstract] OR postterm[Title/Abstract] OR post-term[Title/Abstract] OR stillbirth[Title/Abstract] OR asphyxia[Title/Abstract] OR birth injur*[Title/Abstract] OR birth defect*[Title/Abstract] OR birth complication*[Title/Abstract] OR Congenital, Hereditary, and Neonatal Diseases and Abnormalities[MeSH Terms]

**Filters: publication date 01-01-2007/01-03-2017, languages: Dutch, English**

**Undocumented migrants:**

**Search Pubmed/MEDLINE (25^th^ of April 2017) (search syntax was adapted to Embase database)**

**#1 AND #2**

**#1**

undocumented immigrants[MeSH Terms] OR undocumented[Title/Abstract] OR stateless[Title/Abstract] OR non-citizen[Title/Abstract] OR ((illegal[Title/Abstract] OR unauthorized[Title/Abstract] OR irregular[Title/Abstract]) AND (immigrant*[Title/Abstract] OR migrant*[Title/Abstract] OR women[Title/Abstract] OR mother*[Title/Abstract] OR alien*[Title/Abstract] OR pregnan*[Title/Abstract]))

**#2**
Europe[MeSH Terms] OR Albania*[Title/Abstract] OR Andorra*[Title/Abstract] OR Austria*[Title/Abstract] OR Belarus*[Title/Abstract] OR Belgium[Title/Abstract] OR Bosnia[Title/Abstract] OR Bulgaria*[Title/Abstract] OR Croatia*[Title/Abstract] OR Cyprus[Title/Abstract] OR Czech*[Title/Abstract] OR Denmark[Title/Abstract] OR England[Title/Abstract] OR Estonia*[Title/Abstract] OR Faroe*[Title/Abstract] OR Faeroe*[Title/Abstract] OR Finland[Title/Abstract] OR France[Title/Abstract] OR Germany[Title/Abstract] OR Gibraltar*[Title/Abstract] OR Greece[Title/Abstract] OR Great-Britain[Title/Abstract] OR Great Britain[Title/Abstract] OR Holland[Title/Abstract] OR Hungary[Title/Abstract] OR Iceland*[Title/Abstract] OR Ireland[Title/Abstract] OR Northern Ireland[Title/Abstract] OR Italy[Title/Abstract] OR Kosovo*[Title/Abstract] OR Latvia*[Title/Abstract] OR Liechtenstein*[Title/Abstract] OR Lithuania*[Title/Abstract] OR Luxembourg*[Title/Abstract] OR Luxemborg*[Title/Abstract] OR Macedonia*[Title/Abstract] OR Malta*[Title/Abstract] OR Moldavia*[Title/Abstract] OR Monaco[Title/Abstract] OR Montenegro[Title/Abstract] OR Netherlands[Title/Abstract] OR Norway[Title/Abstract] OR Poland[Title/Abstract] OR Portugal[Title/Abstract] OR Romania*[Title/Abstract] OR San Marino[Title/Abstract] OR Scotland[Title/Abstract] OR Serbia*[Title/Abstract] OR Slovenia*[Title/Abstract] OR Slovakia*[Title/Abstract] OR Spain[Title/Abstract] OR Sweden[Title/Abstract] OR Switzerland[Title/Abstract] OR United Kingdom[Title/Abstract] OR UK[Title/Abstract] OR Ukraine[Title/Abstract] OR Vatican*[Title/Abstract] OR Wales[Title/Abstract] OR Belgian[Title/Abstract] OR Bosnian[Title/Abstract] OR British[Title/Abstract] OR Cypriot[Title/Abstract] OR Danish[Title/Abstract] OR Dutch[Title/Abstract] OR English[Title/Abstract] OR Finnish[Title/Abstract] OR French[Title/Abstract] OR German[Title/Abstract] OR Greek[Title/Abstract] OR Hungarian[Title/Abstract] OR Irish[Title/Abstract] OR Italian[Title/Abstract] OR Kosovar[Title/Abstract] OR Maltese[Title/Abstract] OR Monacan[Title/Abstract] OR Norwegian[Title/Abstract] OR Polish[Title/Abstract] OR Portuguese[Title/Abstract] OR Scottish[Title/Abstract] OR Spanish[Title/Abstract] OR Swedish[Title/Abstract] OR Swiss[Title/Abstract] OR Ukrainian[Title/Abstract] OR Welsh[Title/Abstract] OR Geneva[Title/Abstract] OR Europe*[Title/Abstract] OR EU[Title/Abstract] OR Benelux[Title/Abstract] OR Western Europe*[Title/Abstract] OR Eastern Europe*[Title/Abstract] OR Scandinavia*[Title/Abstract] OR Nordic[Title/Abstract] OR Baltic States[Title/Abstract] OR Balkan[Title/Abstract] OR Mediterranean[Title/Abstract] OR Western countr*[Title/Abstract] OR Destination countr*[Title/Abstract] OR High-income countr*[Title/Abstract] OR High-immigration setting*[Title/Abstract] OR Host countr*[Title/Abstract] OR Immigrant-receiving countr*[Title/Abstract] OR migrant-receiving countr*[Title/Abstract]

**Filter: publication date: 2007-2017**

**File 2. Grey literature search strategy**

**Asylum seekers**

- **Google search**

**For each country/region we searched the first 5 pages:**

- Keywords: asyl pregnant OR pregnancy OR matern “country” filetype:pdf
- Time frame: 01/01/2007 – 25/06/2017
- **Countries/regions searched via Google**

Europe, Albania, Andorra, Austria, Bosnia, Belarus, Belgium, Croatia, Cyprus, Czech Republic, Denmark, England, Estonia, Faroe Islands, Finland, France, Germany, Gibraltar, Greece, Great-Britain, Holland, Hungary, Iceland, Ireland, Northern-Ireland, Italy, Kosovo, Latvia, Liechtenstein, Lithuania, Luxembourg, Macedonia, Malta, Moldavia, Monaco, Netherlands, Norway, San Marino, Scotland, Serbia, Slovenia, Slovakia, Spain, Sweden, Switzerland, United Kingdom, Ukraine, Vatican, Wales, European Union, Benelux, Western Europe, Eastern Europe, Scandinavia, Nordic countries, Baltic countries, Mediterranean countries, destination countries, high income countries, high immigration setting, host countries, migrant-receiving countries

**Undocumented migrants**

- **Google search**

**For each country/region we searched the first 5 pages:**

- Undocumented pregnan outcomes + country/region
- Undocumented matern outcomes + country/region
- **Countries/regions searched via Google**

Europe, Albania, Andorra, Austria, Bosnia, Belarus, Belgium, Croatia, Cyprus, Czech Republic, Denmark, England, Estonia, Faroe Islands, Finland, France, Germany, Gibraltar, Greece, Great-Britain, Holland, Hungary, Iceland, Ireland, Northern-Ireland, Italy, Kosovo, Latvia, Liechtenstein, Lithuania, Luxembourg, Macedonia, Malta, Moldavia, Monaco, Netherlands, Norway, San Marino, Scotland, Serbia, Slovenia, Slovakia, Spain, Sweden, Switzerland, United Kingdom, Ukraine, Vatican, Wales, European Union, Benelux, Western Europe, Eastern Europe, Scandinavia, Nordic countries, Baltic countries, Mediterranean countries, destination countries, high income countries, high immigration setting, host countries, migrant-receiving countries

**File 3. Organisations contacted with request for grey literature**

Individual names of persons contacted are not provided out of privacy considerations

| **Organisation** | **City, Country** | **Region of Focus** |
| --- | --- | --- |
| Landelijk Ongedocumenteerden Steunpunt | NL | NL |
| OKIA | The Hague, NL | NL |
| NIVEL | NL | NL |
| Pharos | NL | NL |
| Stg Lampion (Pharos) | NL | NL |
| GGD | The Hague, NL | NL |
| GGD | Amsterdam, NL | NL |
| Johannes Wier Stichting | NL | NL |
| Wereldhuis | Amsterdam, NL | NL |
| Casa Migrante | Amsterdam. NL | NL |
| INLIA | Groningen, NL | NL |
| Fabel van de Illegaal | Zeist, NL | NL |
| Huize Agnes | Utrecht, NL | NL |
| Wereldvrouwenhuis | Nijmegen, NL | NL |
| Harriet Tubmanhuis | Amsterdam, NL | NL |
| SVZV | Amsterdam, NL | NL |
| KNOV - Tijdschrift voor Verloskundigen | NL | NL |
| Verlokundigenpraktijk Bijlmermeer | Amsterdam, NL | NL |
| Kruispost | Amsterdam, NL | NL |
| Rotterdams Ongedocumenteerden Steunpunt | Rotterdam, NL | NL |
| Pauluskerk | Rotterdam, NL | NL |
| VLOT | Heerlen, NL | NL |
| Moeders van Rotterdam | Rotterdam, NL | NL |
| Defense for Children International | NL | NL |
| Vluchteling onder Dak | Wageningen, NL | NL |
| SNOV | Nijmegen, NL | NL |
| Médecins du Monde | International | EU |
| Médecins du Monde | Austria | EU |
| Médecins du Monde | Belgium | EU |
| Médecins du Monde | Bulgaria | EU |
| Médecins du Monde | Croatia | EU |
| Médecins du Monde | Czech Republic | EU |
| Médecins du Monde | France | EU |
| Médecins du Monde | Germany | EU |
| Médecins du Monde | Greece | EU |
| Médecins du Monde | Hungary | EU |
| Médecins du Monde | Ireland | EU |
| Médecins du Monde | Italy | EU |
| Médecins du Monde | Luxembourg | EU |
| Médecins du Monde | Norway | EU |
| Médecins du Monde | Poland | EU |
| Médecins du Monde | Romania | EU |
| Médecins du Monde | Spain | EU |
| Médecins du Monde | Sweden | EU |
| Médecins du Monde | Swiss | EU |
| Médecins du Monde | Slovenia | EU |
| Médecins du Monde | UK | EU |

**File 4. Request for grey literature**

Dear Sir/Madam,

We are currently conducting systematic literature reviews on mother and infant health among asylum seekers, asylum status holders, and undocumented immigrant women in Europe. These reviews are carried out by a team of researchers from i.a. University Medical Center Utrecht, Dutch Association of Community Health Services & Regional Medical Emergency Preparedness and Planning offices (GGD GHOR) and Médicins du Monde.

Because various actors are active in this field who do not necessarily publish their experiences or studies in peer reviewed journals, we aim to also include 'grey literature', such as reports, articles and guidelines.

 We are reaching out to you because of your or your organization's affinity and/or expertise in health(care) for asylum seekers, asylum status holders, and undocumented migrant women.

In case you have or know of documents we kindly ask you to share this information with us via the forms below. If you happen to know of other organizations or persons we should approach - please let us know using the same form.

1. For sources regarding health of mothers and infants (maternal or perinatal outcomes) among **undocumented migrants** in Europe, please use this form: <https://goo.gl/forms/qWOKar8jaOXEA4cC2>

2. For sources concerning health of mothers and infants (maternal or perinatal outcomes) among **asylum seekers and asylum status holders** in Europe, please use this form: <https://goo.gl/forms/7vMpfPtwWuP1DrS33>

We would like to ask you to share your information by April 30, to allow us for sufficient time to analyze the results. In case you have questions or comments, please contact:

- Undocumented migrants review: Myrthe van Midde (email)

- Asylum seeker review and asylum status holders: Julia Tankink (email)

Thank you very much in advance for your cooperation.

Yours sincerely,

Myrthe van Midde, Julia Tankink and Noor Gieles

On behalf of the review project team:

UMC Utrecht: Dr. Joyce Browne, Dr. Marcus Rijken, Prof. Gouke Bonsel, Prof. Kitty Bloemenkamp, Ms Julia Tankink, Ms Noor Gieles, Ms Mia Wessels

GGD GHOR: Dr. Simone Goosen

LUMC: Dr. Thomas van den Akker

ZGT Almelo: Dr. Peggy  van der Lans

Médecins du Monde: Ms Myrthe van Midde

**File 5. Table 3: Overview of reported outcomes and the direction of findings in each study**

|  | **Asylum seekers (AS) vs. host-country population (HP)** **(unless stated differently)** | | | | | **Undocumented migrants (UM) vs. documented migrants (DM)** **(unless stated differently)** | | | | |
| --- | --- | --- | --- | --- | --- | --- | --- | --- | --- | --- |
| **Mortality-related outcomes** | **N=** | **Higher** | **No difference** | **Lower** | **No comparison** | **N=** | **Higher** | **No difference** | **Lower** | **No comparison** |
| Maternal mortality | 2 | Van Oostrum et al. (2011) |  |  | Van Hanegem et al. (2011) | 0 |  |  |  |  |
| Perinatal mortality | 1 |  | Van Oostrum et al. (2011) |  |  | 3 |  | Wolff et al. (2008); De Jonge et al. (2011) |  | Shortall et al. (2014) |
| Spontaneous abortion/miscarriage | 1 |  |  |  | Kurth et al. (2010) | 1 | Fedeli et al. (2010) *compared to host population and documented migrants* ◊ |  |  |  |
| **Maternal morbidity (pre-birth)** | **N=** | **Higher** | **No difference** | **Lower** | **No comparison** | **N=** | **Higher** | **No difference** | **Lower** | **No comparison** |
| Pre-eclampsia/hypertensive disorders | 0 |  |  |  |  | 2 |  | Wolff et al. (2008) |  | Schoevers et al. (2009) |
| Antenatal depression | 1 |  | Ratcliff et al. (2015) *compared to migrants with non-precarious legal status* |  | Ratcliff et al. (2015)¤ | 0 |  |  |  |  |
| Gestational diabetes | 1 |  |  |  | Kurth et al. (2010) | 1 |  | Wolff et al. (2008) |  |  |
| Anemia during pregnancy | 1 |  |  |  | Kurth et al. (2010) | 1 |  | Wolff et al. (2008) |  |  |
| Cervix insufficiency | 0 |  |  |  |  | 1 |  |  |  | Schoevers et al. (2009) |
| Prenatal bleeding | 1 |  |  |  | Kurth et al. (2010) | 1 |  | Wolff et al. (2008); De Jonge et al. (2011) |  |  |
| Urinary tract infection | 0 |  |  |  |  | 1 |  | Wolff et al. (2008) |  |  |
| Kidney problems during pregnancy | 0 |  |  |  |  | 1 |  |  |  | Schoevers et al. (2009) |
| Risk of preterm birth (unspecified) | 0 |  |  |  |  | 1 |  | Wolff et al. (2008) |  |  |
| Antepartum hospitalisations (unspecified) | 0 |  |  |  |  | 1 | Fedeli et al. (2010)  *compared to host population and documented migrants* ◊ |  |  |  |
| Complications during pregnancy (unspecified) | 0 |  |  |  |  | 1 |  | Wolff et al. (2008) |  |  |
| **Maternal morbidity (birth-related)** | **N=** | **Higher** | **No difference** | **Lower** | **No comparison** | **N=** | **Higher** | **No difference** | **Lower** | **No comparison** |
| Severe acute maternal morbidity | 1 | Van Hanegem et al. (2011) |  |  |  | 0 |  |  |  |  |
| Vaginal tear | 0 |  |  |  |  | 1 |  | Wolff et al. (2008) |  |  |
| Retention of the placenta | 0 |  |  |  |  | 1 |  | Wolff et al. (2008) |  |  |
| Obstetric complications (unspecified) | 1 |  | Ratcliff et al. (2015) |  |  | 1 |  | Wolff et al. (2008) |  |  |
| **Maternal morbidity (post-partum)** | **N=** | **Higher** | **No difference** | **Lower** | **No comparison** | **N=** | **Higher** | **No difference** | **Lower** | **No comparison** |
| Complications post-partum (unspecified) | 0 |  |  |  |  | 1 |  | Wolff et al. (2008) |  |  |
| **Timing of birth** | **N=** | **Higher** | **No difference** | **Lower** | **No comparison** | **N=** | **Higher** | **No difference** | **Lower** | **No comparison** |
| Preterm birth | 1 |  |  |  | Kurth et al. (2010)¤ | 4 | De Jonge et al. (2011) | Wolff et al. (2008) |  | Shortall et al. (2014); Schoevers et al. (2009) |
| Post-term birth | 0 |  |  |  |  | 2 |  | Wolff et al. (2008) |  | Shortall et al. (2014) |
| Gestational age | 0 |  |  |  |  | 2 |  | De Jonge et al. (2011) | Wolff et al. (2008) |  |
| **Mode of birth** | **N=** | **Higher** | **No difference** | **Lower** | **No comparison** | **N=** | **Higher** | **No difference** | **Lower** | **No comparison** |
| Vaginal birth (spontaneous) | 1 |  | Kurth et al. (2010) |  |  | 2 |  | Wolff et al. (2008) |  | Wolff et al. (2008); Shortall et al. (2014) |
| Prolonged labor | 0 |  |  |  |  | 1 |  |  |  | Schoevers et al. (2014) |
| Referral for failure to progress in labor | 0 |  |  |  |  | 1 |  | De Jonge et al. (2011) |  |  |
| Induction of labor | 1 |  | Van Hanegem et al. (2011) *compared to non-Western immigrants and Dutch women with severe acute maternal morbidity* |  |  | 1 |  | Jonge et al. (2011) |  |  |
| Instrumental birth (unspecified) | 0 |  | Kurth et al. (2010) |  |  | 1 |  |  |  | Shortall et al.(2014) |
| Forceps | 0 |  |  |  |  | 2 |  | De Jonge et al. (2011) |  | Wolff et al. (2008) |
| Vacuum | 0 |  |  |  |  | 2 |  | De Jonge et al. (2011) |  | Wolff et al. (2008) |
| Episiotomy | 0 |  |  |  |  | 1 |  | Wolff et al. (2008) |  |  |
| Caesarean section (unspecified) | 1 |  | Van Hanegem et al. (2011) *compared to non-Western immigrants and Dutch women with severe acute maternal morbidity* |  |  | 3 |  | De Jonge et al. (2011) | Fedeli et al. (2010) *compared to host population and documented migrants* | Wolff et al. (2008); Schoevers et al. (2009) |
| Elective caesarian section | 1 |  | Kurth et al. (2010) |  |  | 1 |  |  |  | Shortall et al. (2014) |
| Unplanned/emergency caesarian section | 1 |  | Kurth et al. (2010) |  |  | 1 |  |  |  | Shortall et al. (2014) |
| **Neonatal outcomes** | **N=** | **Higher** | **No difference** | **Lower** | **No comparison** | **N=** | **Higher** | **No difference** | **Lower** | **No comparison** |
| Low birthweight (<2500 grams) | 1 |  |  |  | Kurth et al. (2010) | 4 | De Jonge et al. (2011); Salmasi et al. (2015) *compared before and after acquisition of documented status* | Wolff et al. (2008) |  | Schoevers et al. (2009) |
| Macrosomia | 0 |  |  |  |  | 1 |  | De Jonge et al. (2011) |  |  |
| Retardation of intrauterine growth | 1 |  |  |  | Kurth et al. (2010) | 0 |  |  |  |  |
| Born in good health | 0 |  |  |  |  | 1 |  | Wolff et al. (2008) |  |  |
| Apgar score | 0 |  |  |  |  | 1 |  | Wolff et al. (2008) |  |  |
| Admission to neonatal intensive care unit | 0 |  |  |  |  | 1 |  | Wolff et al. (2008) |  |  |
| Neonatal admission at maternal indication | 0 |  |  |  |  | 1 |  | De Jonge et al. (2011) |  |  |
| Neonatal admission for prematurity/being small-for-gestational age | 0 |  |  |  |  | 1 | De Jonge et al. (2011) |  |  |  |
| Neonatal admission for poor neonatal condition | 0 |  |  |  |  | 1 |  | De Jonge et al. (2011) |  |  |
| Foetal distress | 0 |  |  |  |  | 2 |  | De Jonge et al. (2011) |  | Schoevers et al. (2009) |
| Difficulties in adaptation to extra-uterine life | 1 |  |  |  | Kurth et al. (2010) |  |  |  |  |  |
| Birth trauma | 0 |  |  |  |  | 1 |  |  |  | Schoevers et al. (2009) |
| Mother to child transmission of HIV | 1 |  |  |  | Goosen et al. (2015) |  |  |  |  |  |
| Congenital malformations | 1 |  |  |  | Kurth et al. (2010) | 2 |  |  |  | Schoevers et al. (2009); Shortall et al. (2014) |
| Neonatal complications (unspecified) | 0 |  |  |  |  | 1 |  | Wolff et al. (2008) |  |  |
| **Table description**  All studies were classified as reporting results either higher, lower or similar (no differences) for target populations as compared to host populations/documented migrants, or as not comparing those two.  ◊ Percentages/rates reported only, no statistical analysis conducted  ¤ No control group; national rates are mentioned as a reference (without statistical analysis) | | | | | | | | | | |

**File 6. Risk of bias assessment according to the Newcastle-Ottawa Scale (NOS)**

| **Author(s)** | **Selection (max. 4 stars)** | **Comparability (max. 2 stars)** | **Outcome (max 3 stars)** | **Overall risk of bias** |
| --- | --- | --- | --- | --- |
| **Asylum seekers** | | | | |
| Van Oostrum et al. (2009) | **** | ** | *** | Low |
| Kurth et al. (2010) | **-* | -- | *** | Moderate |
| Van Hanegem et al. (2011) | **** | ** | *** | Low |
| Goosen et al. (2015) | -*** | ** | *** | Low |
| Ratcliff et al. (2015) | -*** | ** | **- | Moderate |
| **Undocumented migrants** | | | | |
| Wolff et al. (2008) | **** | ** | **- | Low |
| Schoevers et al. (2009) | **** | -- | -*- | Moderate |
| Fedeli et al. (2010) | ***- | -- | **- | Moderate |
| De Jonge et al. (2011) | **** | ** | *** | Low |
| Shortall et al. (2014) | --** | -- | -*- | High |
| Salmasi et al. (2015) | **** | ** | *** | Low |

File 7. Additional references (number 41-56)

41. Bakken K, Skjeldal O, Stray-Pedersen B. Higher risk for adverse obstetric outcomes among immigrants of African and Asian descent: a comparison study at a low-risk maternity hospital in Norway. Birth. 2015;42(2):132–40.

42. Minsart A, Englert Y, Buekens P. Naturalization of immigrants and perinatal mortality. Eur J Public Health. 2010;23(2):269–74.

43. Racape J, De Spiegelaere M, Dramaix M, Haelterman E, Alexander S. Effect of adopting host-country nationality on perinatal mortality rates and causes among immigrants in Brussels. Eur J Obstet Gynecol Reprod Biol. 2013;168(2):145–50.

44. Bakken K, Skjeldal O, Stray-Pedersen B. Immigrants from conflict-zone countries: an observational comparison study of obstetric outcomes in a low-risk maternity ward in Norway. BMC Pregnancy Childbirth. 2015;15:163.

45. Liu C, Urquia M, Cnattingius S, Hjern A. Migration and preterm birth in war refugees: A Swedish cohort study. Eur J Epidemiol. 2014;29(2):141–3.

46. Gagnon AJ, Zimbeck M, Zeitlin J. Migration and perinatal health surveillance: An international Delphi survey. Eur J Obstet Gynecol Reprod Biol. 2010;149(1):37–43.

47. Goosen S, Uitenbroek D, Wijsen C, Stronks K. Induced abortions and teenage births among asylum seekers in The Netherlands: analysis of national surveillance data. J Epidemiol Community Health. 2009;63(7):528–33.

48. Wolff H, Lourenço A, Bodenmann P, Epiney M, Uny M, Andreoli N, et al. Chlamydia trachomatis prevalence in undocumented migrants undergoing voluntary termination of pregnancy: a prospective cohort study. BMC Public Health. 2008;8(391):1–7.

49. Casillas A, Bodenmann P, Epiney M, Gétaz L, Irion O, Gaspoz JM, et al. The border of reproductive control: undocumented immigration as a risk factor for unintended pregnancy in Switzerland. J Immigr Minor Heal. 2015;17(2):527–34.

50. Sebo P, Jackson Y, Haller DM, Gaspoz J-M, Wolff H. Sexual and Reproductive Health Behaviors of Undocumented Migrants in Geneva: A Cross Sectional Study. J Immigr Minor Heal. 2011;13(3):510–7.

51. Gagnon AJ, DeBruyn R, Essén B, Gissler M, Heaman M, Jeambey Z, et al. Development of the Migrant Friendly Maternity Care Questionnaire (MFMCQ) for migrants to Western societies: an international Delphi consensus process. BMC Pregnancy Childbirth. 2014;14(1):200.

52. Ingleby D, Petrova-benedict R, Huddleston T, Sanchez E. The MIPEX Health strand : a longitudinal , mixed-methods survey of policies on migrant health in 38 countries. Eur J Public Health. 2018;0(0):1–5.

53. Keygnaert I, Vettenburg N, Roelens K, Temmerman M. Sexual health is dead in my body: participatory assessment of sexual health determinants by refugees, asylum seekers and undocumented migrants in Belgium and the Netherlands. BMC Public Health. 2014;14(416):1–13.

54. Esscher A, Binder-Finnema P, Bødker B, Högberg U, Mulic-Lutvica A, Essén B. Suboptimal care and maternal mortality among foreign-born women in Sweden: maternal death audit with application of the “migration three delays” model. BMC Pregnancy Childbirth. 2014;14(141):1–11.

55. Draper ES, Kurinczuk JJ, Kenyor S. MBRRACE-UK 2015 Perinatal Confidential Enquiry: Term, singleton, normally formed, antepartum stillbirth. The Infant Mortality and Morbidity Studies. Leicester; 2015.

56. Starrs AM, Ezeh AC, Barker G, Basu A, Bertrand JT, Blum R, et al. The Lancet Commissions Accelerate progress — sexual and reproductive health and rights for all : report of the Guttmacher – Lancet Commission. 2018;391.
